# Allogeneic Hematopoietic Stem Cell Transplantation for Adult Patients with Fanconi Anemia

**DOI:** 10.4084/MJHID.2016.054

**Published:** 2016-11-01

**Authors:** Hosein Kamranzadeh Fumani, Mohammad Zokaasadi, Amir Kasaeian, Kamran Alimoghaddam, Asadollah Mousavi, Babak Bahar, Mohammad Vaezi, Ardeshir Ghavamzadeh

**Affiliations:** Hematology, Oncology and Stem Cell Transplantation Research Center; Tehran University of Medical Sciences, Tehran, Iran

## Abstract

**Background and objectives:**

Fanconi anemia (FA) is a rare genetic disorder caused by an impaired DNA repair mechanism which leads to an increased tendency toward malignancies and progressive bone marrow failure. The only curative management available for hematologic abnormalities in FA patients is hematopoietic stem cell transplantation (HSCT). This study aimed to report the results of HSCT in adult or adolescent FA patients.

**Patients and Methods:**

Twenty FA patients with ages of 16 or more who underwent HSCT between 2002 and 2015 enrolled in this study. The stem cell source was peripheral blood, and all patients had a full human leukocyte antigen (HLA) matched donor, 19 patients had a sibling donor, and one had full matched other related. Indications for HSCT were severe bone marrow failure or dependence on blood products transfusion and failure of medical treatment to sustain peripheral blood elements at an acceptable level.

**Results:**

Eleven patients were female and 9 male (55% and 45%). Mean age was 24.05 years. Mortality rate was 50% (n=10), and the leading cause of death was graft versus host disease (GVHD) which occurred in 5 patients (4 cases from acute GVHD and one from chronic GVHD). Survival analysis showed an overall 5-year survival of 53.63% (95% confidence interval: 29.53%–72.74%) and 13 year survival of 45.96 % (95% confidence interval: 22.08%–67.03%) among patients.

**Conclusion:**

HSCT is the only curative management for bone marrow failure in FA patients. But the high rate of mortality and morbidity in adolescent and adult patients makes it a challenging issue.

## Introduction

Fanconi anemia (FA) is a rare inherited disorder characterized by different types of malformations, progressive bone marrow failure and an increased tendency towards both hematological and solid malignancies.^1^ FA arises from an underlying impaired DNA repair mechanism leading to chromosomal instability.^2^

The clinical manifestation of FA varies from case to case; therefore the diagnosis may be delayed until the emergence of cytopenias.^3^ Diagnosis is confirmed with increased chromosomal breakage in cells after exposure to DNA cross-linking agents such as diepoxybutane (DEB) or mitomycin C.^1^

There are some methods available to control the disease as the use of androgens, corticosteroids and supportive care such as transfusion of blood products. But the only curative treatment offered for hematological manifestations of FA is hematopoietic stem cell transplantation (HSCT).^4^–^6^ There are studies available on the outcome of HSCT in FA patients, but most of them are carried out on children. One of the important prognostic factors related to the outcome of HSCT in FA patients is the recipients’ age. It has been shown in some previous studies that advanced age is associated with poorer outcome, more complications and consequently higher mortality.^6^,^7^ One of the most common complications of HSCT is acute GVHD which has a higher incidence in FA patients.^8^ In this study, we focus on outcome and effects of HSCT on adolescents and adults with FA.

## Patients and Methods

All patients with a diagnosis of FA who were 16 years or older and underwent HSCT between 2002 and 2015 were enrolled in this study. The diagnoses were confirmed using mitomycin C sensitivity test which showed increased chromosomal breakage and bone marrow examination was performed including cytogenetic studies. All donors were screened for FA. Covariates are extracted from the patients’ medical records and included age, sex and other basic characteristics, malformations, previous treatments, complete blood counts (CBCs) and bone marrow cellularity, the result of cytogenetic studies, conditioning regimen and acute and chronic GVHD.

Indications for HSCT were severe bone marrow failure or blood product transfusion dependency. Severe bone marrow failure defined as bone marrow cellularity of less than 25% or cellularity of less than 50% with one of the following; absolute neutrophil count (ANC) of less than 500/microliter, platelet count of less than 20000/microliter or reticulocyte count of less than 60000/microliter. All transplants were from a full HLA-matched donor; either sibling or other related, and the source of stem cell was peripheral blood, and none were T-cell depleted. Peripheral blood stem cell (PBSC) was chosen over bone marrow because of feasibility, convenience, and acceptance by donors. Neutrophil engraftment defined as ANC of greater than 500/microliter for 3 consecutive days. No sex limits were set in this study. All diagnostic tests (confirmation of FA), transplants and post-transplant follow up visits took place in hematology and oncology and stem cell transplant research center, Tehran University of medical sciences. Informed consent was obtained from all patients at the beginning of the study.

Continuous variables were presented as mean values, and standard deviations and categorical variables were shown as frequencies and percentages. Overall survival was estimated by the Kaplan-Meier method and accompanied with relevant 95% confidence intervals (CI). Median follow-up time was computed with the reverse Kaplan-Meier method. Univariate analysis of OS to calculate the hazard ratios (HR) of each potential prognostic factors was performed using a Cox proportional hazard regression. A p-value less than 0.05 was considered significant. Data were analyzed using Stata software version 11.2

## Results

Twenty patients participated in our study. Mean age at transplant was 24.05 (SD=8.95) years ranging from 16 to 48 years. 45.0% patients were male (n=9) and 55.0% were female (n=11). The frequency of malformations was 40% (8 cases) whom 6 cases (30.0%) had limb malformations, 1 had nephron-urological malformation, and 1 had urogenital (5.0% each).

Essential characteristics of studied population were briefed in table 1.

Fourteen patients (70%) had severe bone marrow failure, and the remaining 6 (30%) were dependent on transfusion of blood products despite medical treatments.

Based on clinical and pathologic findings there was no evidence of acute leukemia or myelodysplastic syndrome. Cytogenetic studies in all patients revealed no abnormalities.

All patients had a full HLA matched donor (19 siblings and one other related). Seventeen patients received Bu/Cy; Busulfan: 0.2 mg/kg days 9 to day 6 and Cyclophosphamide 15mg/kg days 5 to day 2 before transplantation. And 3 patients received Flu/Cy/ATG; fludarabine: 30mg/m^2^ for 5 days (days 9 to 5 before transplantation), cyclophosphamide 10 mg/kg for 2 days (day 4 and day 3 before transplantation) and equine anti Thymocyte globulin (ATG) 10mg/kg; for days 4 to 1 prior to transplantation as the conditioning regimen. GVHD prophylaxis for Bu/Cy group consisted of methotrexate 10 mg/m^2^ for the first day after transplantation and 6 mg/m^2^ for days 3 and 6, cyclosporine 1.5 mg/kg intravenous days (IV) 3 to 7 after transplantation then 3 mg/kg IV from day 7 until the patient tolerates oral, then changes to 5 mg/kg oral, and folinic acid 15 mg oral days 2, 4, 5, 7, 8 and 9 after transplantation. For the Flu/Cy/ATG group, GVHD prophylaxis was the only cyclosporine as mentioned.

Median follow up time was 26 months (maximum; 159 months).Graft failure occurred only in one patient (5%) and median time to neutrophil engraftment was 11 days after transplant.

Fifteen patients had acute GVHD, 9 patients were grade 1 and six were grades 2 to 4. There were 15 cases of chronic GVHD (9 limited and 6 extensive).

**Figure 1:**
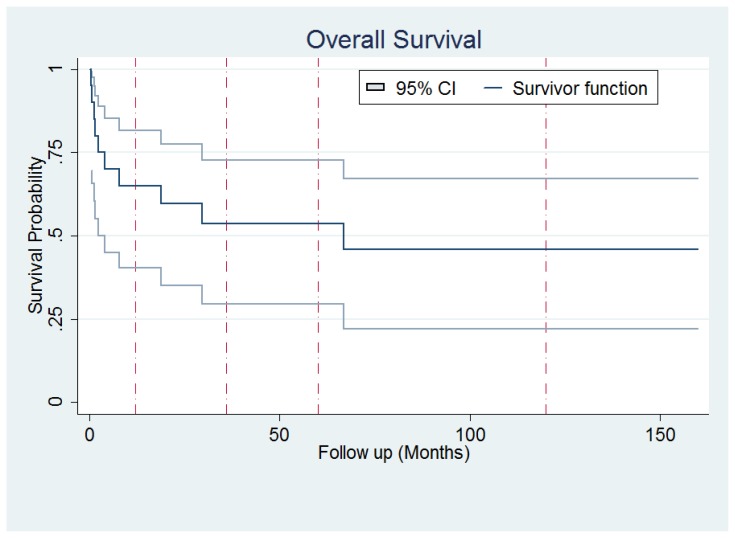
Overall survival of Fanconi Anemia patients.

Mortality rate was 50% (10 out of 20 cases). Survival analysis using Kaplan-Meier estimate were performed and showed a 5-year overall survival of 53.63% (95% confidence interval: 29.53%–72.74%) Figure 1.

The leading cause of death was GVHD with 5 cases; 4 acute and 1 chronic, responsible for 50% of deaths reported.

The most frequent reason for death in the first month was sepsis (1 out of 2 cases died of sepsis, and the other died of sepsis and Diabetic Ketoacidosis; DKA simultaneously) and for the 3-month was acute GVHD. Other causes of death were hepatic mass (a mass lesion with radiologic findings in favor of malignancy but the patient died before the biopsy was taken) and renal failure.

There is a significant relationship between mortality and grade 3 or 4 acute GVHD (log-rank; p-value=0.02).

There were no association between overall survival and age, sex, liver function test, Hb and white blood cell, platelet count and bone marrow cellularity before transplant (cox-regression).

## Discussion

This retrospective study was aimed to assess the outcome of HSCT in adolescents and adults with Fanconi anemia.

Acquired idiopathic aplastic anemia (AA) syndromes are treated with HSCT as well as FA. The report of Passweg et al. on severe AA patients underwent HSCT showed 5-year OS of 66% during 1988–1992.^9^ Also, the report of Sangiolo et al. from Fred Hutchinson have shown 10-year OS of 65% in severe AA patients over the age of 40.^10^ In general, the survival of FA patients is lower than other severe AA conditions. In a multicenter EBMT study in Europe, which analyzed the results of 795 FA patients underwent HSCT from 1972 to 2010, with a median follow up time of 6 years, an overall survival of 49% at 20 years was observed.^7^ However, there are some reports of better OS in transplanted FA patients. Bitan et al who transplanted 7 patients reported a survival of 100% and no severe acute GVHD with a follow up time of 19 to 101 months,^11^ similarly Tan et al in a group of 11 FA patients with aplastic anemia (n=10) or MDS (n=1) showed a 2 year overall and event free survival of 100% and 82% respectively.^12^ These above mentioned studies, performed on small sample size showing a better outcome with a short-term follow-up, include people with a median age at transplant <10 years, before clonal evolution to leukemia/ MDS and, with a conditioning fludarabine based regimens, without radiation.^7^,^12^ Our study is comparable to EBMT results.^7^

Previous cooperative studies performed in a larger number of patients have shown that older age, malformations, and leukemic evolution are associated with worse outcome. In particular Guardiola et al. in a polycentric study on patients with median age at transplantation of 10.8 years (4.0–37.4 years), transplanted from unrelated donors, demonstrated the poor outcome of patients with malformation 5 and the research of Ayas et al., conducted on 3 groups of FA patients with MDS, acute leukemia, and cytogenetic abnormality and a median age of 15, 18 and 13 years old, respectively, demonstrated that younger patients and recipients of HLA-matched related donor transplantations with cytogenetic abnormalities only have the best survival.^6^

These data was fundamentally confirmed by the large EBMT study in which a better outcome was observed for patients transplanted before the age of 10 years, before clonal evolution (i.e., myelodysplastic syndrome or acute myeloid leukemia), from a matched family donor, after a conditioning regimen without irradiation, the latter including fludarabine.^7^ Furthermore, this study stressed the long term negative affect associated with chronic GVHD, more frequent in patients transplanted with PBSC.

The average age in our study was 24.05 years, and we also showed that advanced age is associated to worse outcome which is similar to previous reviews. Acute GVHD was detected in 75% of patients during the follow up time, but most of them (9/15) had grade 1 GVHD. AA secondary to Fanconi should be distinguished from primary AA anemia. Studies report lower incidence in severe AA patients. In Sangiolo et al. avowed that subjects older than 40 years having HCT from HLA-identical siblings had 35% acute GVHD of grade 2 or higher and 26% chronic GVHD at 2-year;^10^ similarly Passweg et al. signaled 19% incidence of acute GVHD grade 2 to 4, and 32% of chronic GVHD, at 5-year respectively in 1988–1992.^9^ Studies on FA patients showed a higher incidence. Peffault de Latour revealed a 32% incidence of grade 2 to 4 acute and 14% and 19% 1-year and 5-year chronic GVHD. Subjects with FA patients seem to be more susceptible to developing acute GVHD, regardless of younger age, rather than non-FA acquired aplastic anemia syndromes in response to HSCT; the Guardiola et al. studies, performed in this field, have shown a relative risk (RR) of 2.00 for FA patients^8^ also a more significant effect of advanced age on GVHD incidence.^8^ The other factor which still remains controversial is the effect of stem cell source, PBSC or bone marrow. Some reports advocate the higher incidence of acute GVHD while some others like a Cochrane review showed only lower acute GVHD grade 3 and 4 with bone marrow, but when grades 2 to 4 are analyzed the difference was not significant.^13^ However, the chronic GVHD seems to be increased.^7^

Our study revealed a significant association between acute GVHD of grades 3 or more and higher mortality. Moreover, other clinically relevant associations, as age and malformations do not reach a significant level from the statistical point of view due to a limited number of patients, typical of data of a rare disease reported by a single-center. Furthermore, in our center, we perform the for Fanconi Anemia only radiation-free therapy conditioning regimens which are shown to be linked to fewer complications such as secondary malignancies without compromising the engraftment.^12^,^14^–^15^ Hence earlier studies suggested without-radiation protocols for conditioning FA patients to be considered.^16^ The rationale for the use of reduced intensity regimens is based on the extreme vulnerability of FA patients to DNA damage which explains better outcomes with RIC regimens and a good immunosuppression, which probably is attained with busulfan and radiation free regimens and with fludarabin and ATG.^19^

The primary cause of mortality in our study was GVHD (4 acute and 1 chronic) and infection which is comparable to former studies.^17^ Guardiola et al. suggested that the effect of on mortality is not confined to severe acute GVHD in the first post-transplant months and it also probably by inducing chronic GVHD can be a risk factor for development of secondary malignancies, particularly in patients transplanted with peripheral blood.^7^,^18^ Experience with a conditioning regimen with fludarabine seems more favorable in term of GVHD and survival.^11^,^12^,^19^ Three of our patients were treated with this regimen, but a confrontation in our set of patients is not possible due to a low number of cases.

The outcome of HSCT in FA patients, in comparison to other idiopathic acquired aplastic anemia syndromes, is less satisfactory due the complications, and a higher mortality and morbidity rate. These complications are more common in olders. It should be considered that HSCT is still the only curative option for hematologic failure in this patients. More studies focusing on new conditioning regimens, better prophylaxis and management of GVHD including the use of different stem cell sources (bone marrow rather than PBSC ) or T-cell depleted methods may be needed to reach better results.

## Figures and Tables

**Table 1:** Basic characteristics of studied population; M means male and F female. Bone marrow cellularity and cell counts are all pretransplant. FMS stands for full matched sibling.

Non N°	Sex	Age Yrs	PMN°/PMN N°/μl	Hb g/dl	Platelets N°/μl	Conditioning Regimen	Bone Marrow Cell %	Malformation Site	Acute GVHD grade	Chronic GVHD Chronic Grade/severity	State Outcome Status	Donor Dono Donor	Time Survival Months
1	M	18	1500	8.9	10000	Bu/Cy	60	Limb	1	Extensive/severe	Dead	FMS	28
2	F	22	350	7.0	8000	Bu/Cy	60	None	1	None	Alive	FMS	41
3	F	25	800	9.5	25000	Bu/Cy	15	Limb	1	Extensive/severe	Alive	FMS	91
4	M	29	550	7.4	15000	Bu/Cy	25	None	1	Limited/mild	Alive	FMS	95
5	F	26	350	9.2	18000	Bu/Cy	15	GU	1	None	Dead	FMS	2
6	M	33	350	8.4	25000	Bu/Cy	15	None	3	Limited/mild	Dead	FMS	67
7	F	41	150	7.9	9000	Bu/Cy	15	None	0	None	Dead	FMS	<1
8	M	18	500	7.1	20000	Bu/Cy	25	None	1	None	Dead	FMS	<1
9	F	19	500	8.4	25000	Bu/Cy	10	None	1	Limited/mild	Alive	Related	11
10	M	18	1000	6.0	40000	Flu/Cy/ATG	40	Limb	0	Limited/mild	Alive	FMS	160
11	F	16	550	7.6	11000	Flu/Cy/ATG	10	None	1	Extensive/mild	Dead	FMS	19
12	F	17	150	9.5	25000	Flu/Cy/ATG	25	None	0	Limited/mild	Alive	FMS	104
13	F	16	1150	10.6	14000	Bu/Cy	5	Kidney	0	Limited/mild	Alive	FMS	96
14	F	19	600	5.5	14000	Bu/Cy	50	None	3	Extensive/severe	Dead	FMS	1.5
15	M	34	2200	7.9	37000	Bu/Cy	50	Limb	2	Extensive/moderate	Dead	FMS	8
16	M	48	1950	8.4	8000	Bu/Cy	10	None	0	Limited/mild	Alive	FMS	82
17	F	26	1350	8.9	53000	Bu/Cy	25	None	3	None	Dead	FMS	1.3
18	F	22	200	8.2	4000	Bu/Cy	90	Limb	3	Limited/moderate	Dead	FMS	4
19	M	16	1800	9.4	25000	Bu/Cy	5	None	2	Extensive/severe	Alive	FMS	24
20	M	18	200	8.2	87000	Bu/Cy	5	Limb	1	Limited/mild	Alive	FMS	60
